# Recent advances in HIV-1 envelope-based vaccine designs for guiding broadly neutralizing antibody response

**DOI:** 10.1128/jvi.02202-25

**Published:** 2026-04-21

**Authors:** Swarandeep Singh, Sanjeev Kumar, Kalpana Luthra

**Affiliations:** 1 Department of Biochemistry, All India Institute of Medical Sciences 28730, New Delhi, India; 2 Emory Vaccine Centre Program, International Centre for Genetic Engineering and Biotechnology 28845, New Delhi, India; Indiana University Bloomington, Bloomington, Indiana, USA

**Keywords:** HIV-1 vaccine platforms, envelope glycoprotein, broadly neutralizing antibodies, structure guided vaccine design, germline targeting immunogens

## Abstract

Despite decades of intensive research, a protective prophylactic HIV-1 vaccine remains elusive. Considerable efforts have been made in developing HIV-1 vaccines which are capable of neutralizing diverse clades by utilizing conserved epitopes on the HIV-1 envelope glycoprotein (Env). However, traditional Env immunogens typically elicit only clade-specific neutralizing antibodies (nAbs), underscoring the need for novel strategies to trigger rare B cell precursors and then steer affinity maturation toward broadly neutralizing antibody (bnAb) induction. In recent years, diverse innovative vaccine antigen design strategies have been explored to induce protective bnAb response. In this review, we discuss the key technological advances in Env-based vaccine design, emerging immunization strategies, and insights from the recent clinical trials. It further provides a unique perspective on pediatric/infant-derived immunogen strategies. Ongoing collaborative efforts in rational immunogen design, optimized delivery strategies, and strategic immunization regimens will be critical for developing a vaccine capable of eliciting durable protective bnAb responses against HIV-1.

## INTRODUCTION

Despite more than 40 years of concerted research efforts, the HIV-1 pandemic has continued to infect millions of individuals and remains a major global health problem (1). While researchers continue to work toward the development of an effective HIV-1 vaccine, substantial progress has been made in controlling the disease over the past two decades. The widespread use of combination antiretroviral therapy (cART) has markedly lowered the incidence of new infections and improved the life expectancy and quality of life of people living with HIV-1 (PLHIV) (2, 3). Although cART effectively controls HIV-1 in PLHIV, it does not provide a cure for HIV-1 infection and requires lifelong adherence, may cause side effects, and cannot fully prevent long-term comorbidities, highlighting the urgent need for a prophylactic vaccine (4, 5).

Given that neutralizing antibodies (nAbs) represent the key correlate of protection for most licensed antiviral vaccines, numerous HIV-1 vaccine strategies have been explored to elicit protective nAb responses (6–8). These efforts are further motivated by the observation that approximately 10%–25% of HIV-1-infected individuals naturally develop broadly neutralizing antibodies (bnAbs), capable of neutralizing a diverse spectrum of viral clades (9). However, during natural infection, these bnAbs are often outnumbered by the circulating non-neutralizing antibodies (non-nAbs); hence, aviremic individuals are rare (10). A rare group of HIV-1-infected individuals, elite neutralizers (ENs), who develop exceptionally potent bnAbs, could provide valuable blueprints for rational HIV-1 immunogen design (11). Over the past decade, technological advances in single B-cell isolation, high-throughput neutralization screening, computational and structural biology have enabled the discovery and characterization of more than 200 second-generation potent HIV-1 bnAbs (12–14). The availability of stabilized envelope (Env) trimers from diverse clades, combined with high-resolution structural data, has provided detailed insights into the molecular determinants of bnAb recognition (15–18). Leveraging this knowledge by applying the Reverse Vaccinology 2.0 approach or structure-guided immunogen design has brought the goal of developing an HIV-1 vaccine capable of eliciting nAbs within reach (19).

This review summarizes recent developments in the design of HIV-1 envelope (Env)-based immunogens and the strategies being explored to engage rare precursor B-cells and elicit broadly neutralizing antibodies (bNAbs). We discuss emerging immunization approaches, insights from recent clinical trials, and technological advancements that have made available high-resolution structures of engineered stable and native-like HIV trimers that are driving the field forward. Here, we address the challenges associated with these strategies and discuss possible future directions required to develop a vaccine capable of eliciting potent cross-reactive neutralizing antibody responses against HIV-1.

## HIV-1 ENVELOPE gp160 IS A PRIME TARGET OF BROADLY NEUTRALIZING ANTIBODIES (bnAbs)

**Fig 1:**
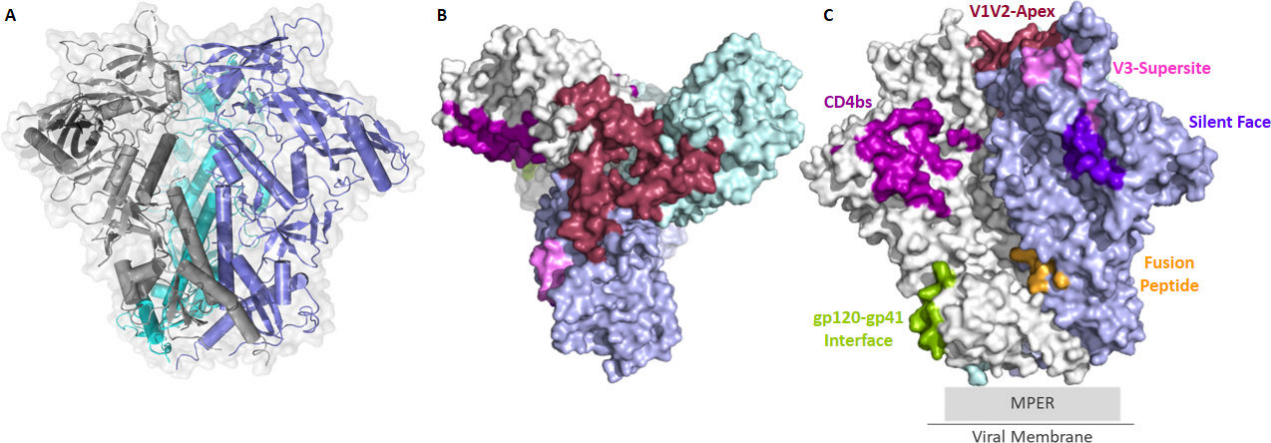
(**A**) Structural representation of HIV-1 envelope trimer- Envelope promoters highlighted in blue, cyan, and gray color. (**B**) Top view of HIV-1 envelope trimer highlighting major HIV-1 bNAb epitopes. (**C**) Surface representation of side view of an HIV-1 envelope trimer highlighting major antibody hotspots: raspberry—V1V2 Apex, violet—V3 Supersite, purple blue—Silent Face, purple—CD4bs, green—gp120-gp41 Interface, orange—Fusion peptide. The structure was generated using the crystal structure of BG505 SOSIP.664 (PDB ID: 4ZMJ) highlighted with the program PyMOL (http://www.pymol.org/).

Natural HIV-1 infection induces a broad spectrum of antibodies including non-nAbs and nAbs (20). Non-nAbs target multiple viral proteins, and their contribution to antiviral defense is mainly through Fc-mediated effector functions such as antibody-dependent cellular cytotoxicity (ADCC), antibody-dependent cellular phagocytosis (ADCP), and complement-dependent cytotoxicity (CDC) (21–23). The nAbs, however, are directed exclusively against the Env glycoprotein, where they block receptor or co-receptor engagement, interfere with viral fusion, or destabilize Env, thereby preventing the conformational changes required for fusion and leading to direct viral neutralization (20, 24, 25). The HIV-1 Env protomer is synthesized as a gp160 precursor in the rough endoplasmic reticulum and is further transported to the Golgi complex where the cellular furin-like protease cleaves it into gp120 and gp41 (26). HIV-1 accommodates up to ~8–10 Env trimers per virion, which are integrated into the lipid membrane, and each complex is composed of heterodimeric trimers of a surface glycoprotein gp120 and a transmembrane spanning protein gp41, that are non-covalently linked to each other (Fig. 1A) (27, 28). The gp120 unit mediates attachment of the virus to the target cell, while the gp41 drives the fusion of virus and target cell membranes (29). The HIV-1 Env is a dynamic structure that shifts from a pre-fusion to a post-fusion state; interestingly, the nAbs are mainly generated against the pre-fusion closed conformation of the Env (30). HIV-1 consistently evades the host immune response through a range of strategies, including rapid, error-prone mutations that create extensive sequence diversity and structural features of its envelope, including positioning highly variable loops in gp120 that conceal conserved epitopes from immune recognition and formation of narrow pockets that limit nAb accessibility to key regions (31–34). Steric hindrance further limits antibody access to the coreceptor-binding site and conformational masking protects neutralization-sensitive regions (35, 36). An evolving, dense glycan shield helps the virus escape immune surveillance, while nonfunctional Env forms, such as gp120/gp41 monomers and gp41 stumps, divert the B-cell responses away from native trimers (37–39). Finally, the low density of Env spikes impairs cross-linking of bivalent nAbs, reducing B-cell activation and survival signaling (40).

Despite the above-mentioned obstacles, the human immune system has the ability to induce Env-specific bnAbs after a certain period of infection in select individuals (13). These are a special class of nAbs that can neutralize a broad spectrum of global HIV-1 clades (13, 41). A fraction of bnAbs can emerge in approximately 10%–20% of HIV-1-infected people that target several independent sites on the viral envelope glycoprotein (42). These bnAbs primarily focus on seven conserved regions on the HIV-1 Env highlighted in Fig. 1B and C: the V1V2-apex, V3-glycan patch, CD4-binding site (CD4bs), gp120–gp41 interface, membrane-proximal external region (MPER), fusion peptide, and the silent-face center, identifying these epitopes as potential targets for vaccine design (12, 25, 43). It is widely anticipated that sustained titers of potent bnAbs would confer protection against diverse HIV-1 clades (12, 13). A key objective of HIV-1 vaccine development is to elicit such protective bnAbs through vaccination. In this review, we have highlighted and discussed the diverse strategies that are being explored to design immunogens capable of presenting key epitopes that could drive an efficacious nAb response upon vaccination.

## STRATEGIES TO DESIGN ENVELOPE-BASED IMMUNOGENS

A wide range of Env protein variants have been tested over the years as vaccine candidates to induce nAbs. Herein, we review these approaches, starting with the simplest Env-derived immunogens, the gp120 env constructs, then progressing through native-like trimers, consensus-based design, multivalent antigen presentation, and vector-based deliveries.

**Fig 2:**
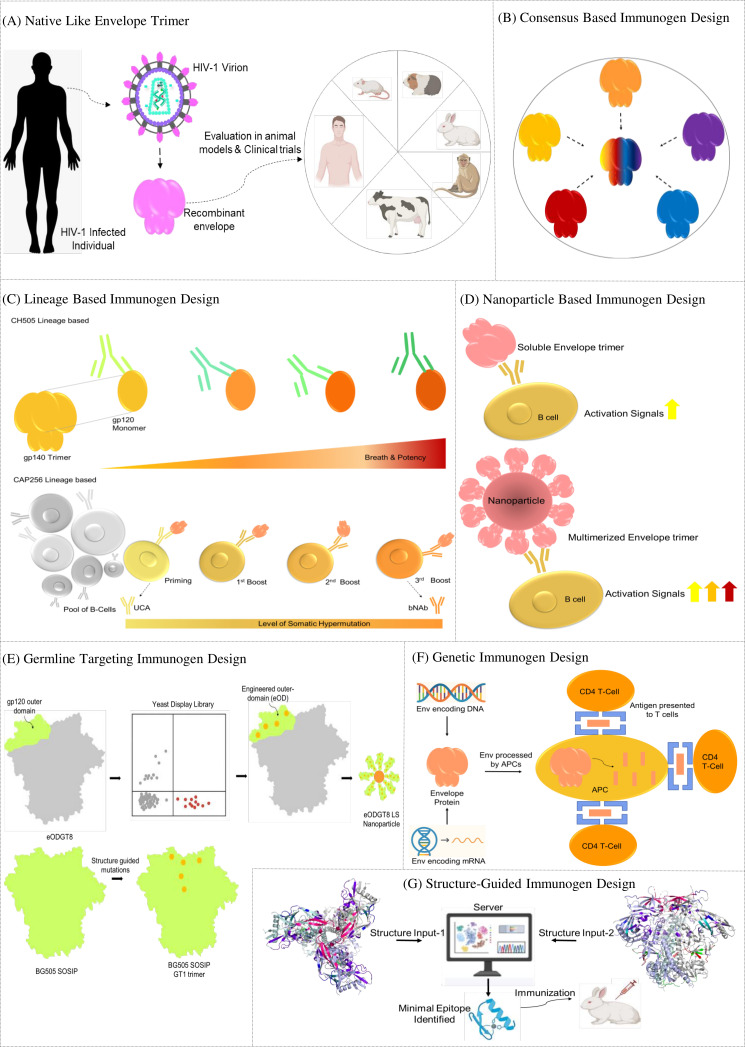
Schematic representation of various Env-derived immunogen design approaches aimed at eliciting broadly neutralizing antibodies: (**A**) design of native-like trimeric Env constructs, (**B**) consensus-based immunogen design, (**C**) lineage-based immunogen design, (**D**) nanoparticle-based immunogen design, (**E**) germline-targeting immunogen design, (**F**) genetically engineered immunogen design, and (**G**) structure-guided immunogen design.

Furthermore, we have provided insight of more complex strategies like the lineage-based and germline targeting immunogen designs and the upcoming mRNA platforms, concluding with recent clinical trials of these constructs. A schematic representation of the different Env immunogen designs is depicted in Fig. 2.

Early vaccine efforts focused on gp120 monomers as immunogens, but these constructs failed to induce nAbs against resistant Tier-2 viruses and showed no protection in Phase III trials (Vax004 in North America and Vax003 in Thailand), with infection rates reaching 6.7% among the vaccine recipients (rgp120 HIV Vaccine Study) (44, 45). The poor efficacy observed was largely due to several structural and immunological issues, largely because key quaternary epitopes recognized by the bnAbs were missing; furthermore, the V3 loop was structurally unstable (46, 47). As a result, the immune response was mainly directed toward non-neutralizing epitopes. These limitations made it clear that a more native-like design was needed, leading to the development of trimeric Env constructs that include both gp120 and gp41 subunits to better display the epitopes targeted by bnAbs. Initial attempts to create soluble Env proteins centered on gp140, which was produced by introducing a stop codon at position 664 and removing the gp41 region before the transmembrane and cytoplasmic tail domains (48, 49). This approach, however, disrupted the stability of the Env trimer, thereby causing gp140 to largely exist as monomers. This hurdle was overcome by replacing the natural REKR cleavage site with a non-cleavable SEKS, covalently linking gp120 to the gp41 ectodomain and generating uncleaved gp140 trimers (50). Trimerization was further promoted by attaching external stabilizing motifs such as GCN4, Fibritin, T4 foldon, or isoleucine zipper to the C-terminus of gp41 (50, 51). Despite these modifications, structural studies revealed that the resulting uncleaved trimers show little resemblance to the native-like Env spike. Rather than forming stable trimers, these constructs mostly broke down into a mix of monomers, dimers, and unstable aggregates caused by incorrect disulfide linkages (52–54). When tested in immunization studies, they failed to generate cross-neutralizing antibody responses in both animal models and human participants (55–57). Taken together, these results clearly showed that the uncleaved gp140 molecules did not function well as vaccine candidates, highlighting the necessity of creating cleavage-competent Env mimics that more accurately reflect the structure of the native viral spike.

## NATIVE-LIKE ENVELOPE TRIMER DESIGN

A significant breakthrough in HIV-1 vaccine development has been the generation of native-like Env trimers with the introduction of two widely studied platforms, SOSIP.664 and the Native Flexible Linker (NFL), across the different HIV-1 clades (Fig. 2A) (58, 59). In both approaches, a stop codon was placed at residue 664 of the envelope protein, truncating the hydrophobic MPER region and generating soluble gp140 Env proteins (60). In the SOSIP platform, trimer instability was addressed by placing a disulfide bond (501C-605C) linking gp120 to gp41 (SOS) and an isoleucine-to-proline substitution (I559P) in gp41, to lock the protein in its prefusion conformation (61, 62). To enhance proteolytic processing of the Env precursor, the native furin cleavage site (REKR) was substituted with a hexa-arginine motif (RRRRRR), resulting in more efficient cleavage between gp120 and gp41 (63). Incorporation of this and other stabilizing mutations into the clade A BG505 Env sequence led to the development of the first well-defined, stable, native-like Env trimer, BG505 SOSIP.664 (15, 16). Incorporation of this and other stabilizing mutations into the clade A BG505 Env sequence led to the development of the first well-defined, stable, native-like Env trimer, BG505 SOSIP.664 (64). The NFL design strategy offers an alternative Env design by replacing the furin cleavage motif with a flexible glycine-serine linker that covalently links gp120 to gp41 while retaining the IP mutation to improve trimer formation (59). This approach avoids the need for furin overexpression, is transferable across HIV-1 clades, and produces trimers that are structurally and antigenically similar to BG505 SOSIP.664. Structural analyses have shown that NFL trimers adopt native-like conformations and immunization studies revealed their ability to elicit autologous neutralizing responses (65).

Over the past few years, multiple stabilizing mutations have been discovered, aimed to enhance Env expression and trimerization. The early base modifications in the SOSIP platform were designated as v2 (version 2), which included incorporation of 501C-605C, 559P, R6, and ΔMPER to improve prefusion stability (58). Subsequent modifications, v3, included 553M and 543Q/N, while v4 incorporated E64K, A66R, and A316W, to reduce exposure of V3-directed non-neutralizing epitopes (66); v5 introduced additional disulfide bonds (72C-564C and 73C-561C) and v6 added 49C-555C to further constrain trimer flexibility (67). Guenaga et al. described BG505 trimer-derived TD8 mutations (47D, 49E, 65K, 106T, 165L, 429R, 432Q, 500R), which significantly stabilized 16,055 & JRFL trimers (68). Steichen et al. reported MD39 mutations (519S, 568D, 570H, 585H) that increased expression and Env trimerization (69). Recently, Del Moral-Sánchez et al. defined v9 mutations (304V, 319Y, 519S, 568D, 570H, 585H) to further enhance trimerization propensity (70). To stabilize the Env trimers in the prefusion closed conformations, Rutten et al. described the repair and stabilize (RnS) mutations (647F, 651F, 655I, & 658V) that are transferable across diverse HIV-1 clades (71). More recently, Rawi et al. described the automated Env stabilization pipeline, which combines Env sequence repair by identification and substitution of rare residues with consensus residues, followed by structure-guided stabilization and introduction of DS-SOSIP intra-gp120 disulfide bond (201C-433C) (72, 73). These series of modifications have enabled the production of a wide range of native-like soluble Env trimers across multiple HIV-1 clades (58, 68, 74–79). Although monovalent immunization of native-like trimers elicits strong autologous Tier-2 neutralizing responses in animals, heterologous neutralization remains limited (64, 80), which is plausibly due to the complex maturation pathways required to develop neutralizing breadth. Consequently, more precise immunogen designs and vaccination strategies are warranted to fully harness the potential of these trimers for effective HIV-1 vaccines.

## PRIMING IMMUNOGENS: GERMLINE TARGETING VACCINE DESIGN

Germline-targeting (GT) immunogen design has emerged as a promising strategy to initiate bNAb lineages against HIV-1 by engaging their inferred naïve B-cell precursors. These germline B cells represent the earliest members of an antibody lineage, but early studies demonstrated that inferred bNAb precursors generally lack measurable affinity for wild-type Env proteins, explaining why conventional immunogens rarely activate them (81). This insight led to the development of engineered germline-targeting immunogens specifically designed to bind such rare precursor B cells and initiate their expansion. The primary goal of GT priming is to select, diversify, and expand these rare clones that have the potential to mature into bnAbs. Several strategies have been employed in the development of GT immunogens. Early research focused on glycan modification approaches, including GT constructs such as 426c.TM4ΔV1-V3 (82). Structure-guided designs further led to the engineered outer domain immunogens such as eOD-GT6 and the optimized eOD-GT8 60mer (Fig. 2E) (83). More recently, trimer-based platforms have also been advanced, including BG505 SOSIP.v4.1-GT1, and the next-generation MD39-11MUTB Env trimer, which was designed to engage glycan-dependent precursors of the PGT121 class (69, 84).

Mascola and colleagues investigated the influence of N-linked glycosylation sites within Loop D and the V5 region of gp120, which are known to modulate the neutralizing activity of VRC01-class antibodies (85). Guided by structural studies, they engineered the clade C gp120-based immunogen 426c. TM4ΔV1-V3, in which the glycan at position 276, being a major barrier to germline antibody binding, was removed, thereby enhancing recognition by both mature and germline-reverted forms of NIH45-46 and other VRC01-class antibodies (82). While this construct demonstrated improved engagement of VRC01 precursors, a key limitation was that the CD4 binding site was not stabilized in its native trimeric conformation, inadvertently exposing non-neutralizing epitopes and potentially diverting immune responses away from the desired bnAb specificities. In another study, Jardine and colleagues developed the engineered outer domain immunogen eOD-GT8, based on the HIV-1 gp120 Env, designed to bind both mature VRC01-class bnAbs (VRC01, 3BNC60, and PGV04), and their inferred germline precursors (83). Furthermore, eOD-GT8 was optimized by multivalent display on nanoparticles (described in detail under the multivalent antigen section) to enhance the activation of the VRC01-class naïve B cells *in vivo*. Immunization with eOD-GT8 knock-in mice expressing germline-reverted VRC01 heavy chains successfully induced antibodies showing characteristics of the VRC01-class bnAbs, with short CDRL3, demonstrating its ability to selectively bind and activate specific VRC01 class precursor BCRs (86). A proof of concept for germline targeting vaccines, based on immunization of AS01B adjuvanted eOD-GT8 60mer, has shown to induce VRC01-class bnAb precursors in 97% of vaccine recipients in the IAVI G001 phase I clinical trial (87). Subsequent studies demonstrated that priming with adjuvanted eOD-GT8, followed by heterologous boosting (HXB2) with rationally designed protein and mRNA-based immunogens (core-g28v2), incorporating iterative rounds of structural remodeling, resurfacing, and glycan masking on nanoparticle scaffolds, can drive antibody maturation toward VRC01-class bnAb development in humanized mice, thereby supporting the initiation of the IAVI G002 phase I clinical trial, evaluating an mRNA-encoded nanoparticle vaccine (88).

Another promising target for GT-immunogen design is the trimer apex. Apex-directed antibodies, derived from multiple germline genes, tend to emerge relatively early and frequently during infection (89, 90). Since these bnAbs recognize quaternary V2 epitopes on the env trimer, trimer-based immunogens are likely required to initiate their lineages. The BG505 SOSIP.v4.1-GT1 trimer was designed to engage germline bnAbs targeting both the trimer apex and the CD4 binding site (84). This construct incorporated several modifications, such as the elimination of N276 and N462 glycosylation sites to remove steric hindrance for CD4bs engagement, and substitutions T278R and G471S to improve contacts with the VRC01 light chain. When GT1 trimers were tested in knock-in mice, the immunizations elicited antibodies with binding characteristics consistent with the VRC01-class germline precursors, indicating successful activation of the target naive B cells *in vivo* (84). However, subsequent boosting with optimized immunogens remains necessary to drive SHM in these precursors and guide their maturation to achieve sufficient breadth and potency.

Using information gathered from studies on mammalian cell surface display coupled with structure-guided directed evolution and random mutagenesis, Steichen et al. engineered stabilized HIV Env trimers with affinity for inferred germline precursors of the PGT121-class bNAbs (69). Starting with WT BG505 N332 Env, successive design intermediates (MD33) culminated in the MD39-11MUT_B variant, which maintained native-like structure and exhibited detectable binding to multiple germline-reverted PGT121 variants. When presented as multimerized liposome-conjugated trimers, these germline-targeting constructs activated PGT121 inferred-germline B cells *ex vivo* and primed PGT121-like serological responses in the PGT121 inferred-germline knock-in mice, illustrating the potential of germline-targeting Env immunogens for priming bnAb precursor B cells. Furthermore, their group designed N332 germline-targeting (N332-GT) Env trimers using BG18-lineage–guided directed evolution and multi-target optimization, generating a series of immunogens (N332-GT1, -GT2, and -GT5), with progressively increased affinity for and improved priming of BG18-class precursor B cells (91). Notably, N332-GT5 formulated with Saponin/MPLA NPs elicited BG18-class precursors in immunized macaques and activated diverse BG18-class HCDR3 B-cell lineages within both the germinal center and memory B-cell pools (92). These findings highlight the potential of this platform as a starting point for sequential vaccination strategies aimed at guiding early B cell responses toward the development of bnAbs. The targeting of the germline antibody precursors is now acknowledged as an important strategy for HIV-1 Env vaccines that are intended to induce bnAbs in humans. These studies propagated prime-boosting strategies, in which a GT prime is to be followed by a boost/s, with progressively less modified design intermediates and then with mature envelope/s, to achieve sufficient SHMs, leading to the production of bnAbs.

## PEDIATRIC OR INFANT-DERIVED Env IMMUNOGENS

The development of bnAbs in HIV-1-infected pediatric or infant ENs provides critical insights into the co-evolution of the virus and bnAbs, offering a valuable framework for HIV-1 vaccine design (93, 94). Longitudinal studies have demonstrated that, unlike adults who typically require several years to develop bnAbs, infants and children can generate potent bnAbs, as early as 1 year post-infection (93, 94). Plasma bnAbs in infant elite and broad neutralizers have been observed to predominantly target the V2-apex region of the HIV-1 Env, and multivariant infection is often associated with the emergence of plasma bnAbs that are also effective in neutralizing diverse autologous viruses (95). This rapid bnAb development is associated with specific viral characteristics, such as the presence of diverse circulating viral variants that provide continuous antigenic stimulation and drive the maturation of cross-neutralizing antibody responses (96, 97). One such example is BG505 Env, derived from a Clade A infant transmitted founder virus, that has been extensively studied over the last decade and is currently being evaluated in vaccine clinical trials (Table 1). A recent study evaluating the germline-targeting (GT) immunogen BG505.GT1.1 in juvenile and infant rhesus macaques observed higher-magnitude neutralizing antibody responses in the infant macaques, suggesting that early-life vaccination with GT immunogens could better promote bnAb development and form the basis for the development of pediatric HIV-1 vaccine platforms (98). It has been established that single Env-based regimens are unlikely to generate bnAb responses, sequential immunizations using multiclade Env cocktails could offer more promise. Notably, the global predominance of clade C, which is responsible for over half of new infections worldwide, underscores the strategic need to design clade C-specific immunogens (99). Recently, we have reported a unique pair of monozygotic twins (AIIMS_330 & AIIMS_329) as pediatric elite neutralizers and engineered and stabilized clade-C soluble Env trimers (330 SOSIP/NFL & 329 SOSIP) and multivalent ferritin/two-component I53-50 nanoparticles (77, 96, 100). These trimers exhibited native-like conformations, preserved multiple bnAb epitopes, and elicited immunogenicity in rabbit models. Such stabilized trimers can function as antigenic baits to isolate novel bnAbs and could inform clade C-based Env vaccine design. Priming with infant/pediatric-derived GT immunogens in a polyvalent vaccine platform holds promise for inducing protective immune responses that are both broad and potent, aligning with the viral diversity encountered in endemic regions.

**TABLE 1:** HIV-1 envelope vaccine candidates in ongoing and recently completed clinical trials^*a*^

S. no.	Trial phase	Trial identifier	Platform	HIV-1 Env immunogen	Adjuvant	R.O.A.	Env dose	Prime & boost (months)
1	Phase I	NCT03699241	Recombinant Protein	BG505 SOSIP.664 gp140	Alum	I.M.	Env-30 μg, 100 μg, 300 μg	0, 2, 4, 8, 12
2	Phase I	NCT04224701	Recombinant Protein	BG505 SOSIP GT1.1 gp140	MPLA-L	I.M.	Env-30 μg, 300 μg	0, 2, 6
3	Phase I	NCT05772286	Recombinant Protein	763 SOSIP v8.2 gp140	MPLA-L	I.M.	Env-100 μg	0, 8, 24, 48
4	Phase I	NCT03961438	Recombinant Protein	ConM SOSIP.v7 gp140	MPLA-L	I.M.	Env-100 μg	0, 2, 6
5	Phase I	NCT04985760	Recombinant Protein	Trimer 4571 (DS-SOSIP)	Alum	I.M.	Env-100 μg	0, 8, 20
6	Phase I	NCT05471076	Recombinant Protein	426c.Mod.Core-C4b & BG505 SOSIP.GT1.1 gp140	3M-052-AF + Alum	I.M.	Env-30 μg, 100 μg, 300 μg	0, 8, 24, 40
7	Phase I	NCT06033209	Recombinant Protein	BG505 N332-GT5 gp140	Saponin/MPLA- NP	I.M., S.C.	N.A.	0, 8, 10
8	Phase I	NCT06796686	Recombinant Protein	426c.Mod.Core-C4b, HxB2.WT.Core-C4b	3M-052 AF + Alum	I.M.	Env-550 μg, 1,100 μg	N.A.
9	Phase I	NCT01423825	Recombinant Protein	CN54 V2 deleted gp140	MF59C.1	I.M.	Env-100 μg	0, 3
10	Phase I	NCT01423825	Recombinant Protein	CN54 V2 deleted gp140	MF59C.1	I.M.	Env-100 μg	0, 3
11	Phase I	NCT05208125	Viral vector & protein based	ChAdOx1.HTI & MVA.HTI, ConM SOSIP.v7 gp140	MPLA-L	I.M.	Env-100 μg	0, 4, 12, 28
12	Phase I	NCT03408262	Viral vector & protein based	Ad4-EnvCN54 & MVA-CN54, CN54gp140	MPLA-L	I.M.	Env-100 μg, Vector-1 × 10¹⁰ VPs	0, 3, 6
13	Phase I/IIa	NCT02315703	Viral vector & protein based	Ad26.Mos.HIV & MVA-Mosaic, gp140	Aluminum phosphate	I.M.	Env-50 μg, 250 μg, Vector-5 × 10¹⁰ VPs	0, 12, 24, 48
14	Phase I	NCT05182125	Viral vector & protein based	AdC6-HIVgp140 and AdC7-HIVgp140, CH505TF gp120	GLA-SE	I.M.	Env-400 μg, Vector-1 × 10¹⁰ VPs	0, 3, 6
15	Phase I	NCT06332339	Viral vector & protein based	16,055 NFL Delta Gly4 Env, Trimer 4571 & Ad4-Env145NFL viral particles	3M-052-AF + Alum	I.M., I.N.	Env-100 µg and 200 µg, 5 × 10⁸ VPs	0, 2, 4, 8, 12
16	Phase I	NCT05828095	DNA based	DNAs-Env Trimer-4571, Interleukin-12 (INO-6160)	3M-052-AF + Alum	Env-I.M., IL-I.D.	Env-100 μg, IL-2-mg	0, 1, 3, 6
17	Phase II	NCT04066881	DNA, viral vector & protein based	DNA-HIV-PT123 (ZM96 Gag, ZM96 Env & CN54 Pol-Nef) & AIDS VAX gp120 B/E, DNA-HIV-PT123 & CN54gp140, MVA & CN54gp140	MPLA-L	I.M.	Env-100 μg, DNA- N.A., Vector-1 × 10⁸ PFU	0, 4, 24, 48
18	Phase I	NCT04844775	DNA & Protein based	Drep-HIV-PT1 (HIV-1 gp140 (96ZM651)) & CN54 gp140, DNA-HIV-PT123 (9ZM96 Gag, ZM96 Env & CN54 Pol-Nef) & CN54 gp140	MPLA-L	I.M.	Env- 500 μg, DNA-0.2 mg, 1 mg, 4 mg	0, 4, 9, 26
19	Phase I	NCT06694753	mRNA nanoparticle based	mRNA-1645-eODGT8 & mRNA-1645-CoreG28v2 & mRNA-1645-N332GT5	N.A.	I.M.	Env-10 μg, 30 μg	0, 8
20	Phase I	NCT05903339	Ferritin NP & mRNA LNP	V3G CH848 Pr-NP1 & CH848 mRNA-Tr2	3M-052 AF + Alum	I.M.	Env NP-60 µg and 100 µg, mRNA-50 µg	0, 2, 6, 10
21	Phase I	NCT06919016	RNA based	DV700P-RNA, DV701B1.1-RNA	N.A.	I.M.	50 µg, 100 µg, 150 µg	0, 8, 24, 40
22	Phase I	NCT06557785	mRNA LNP	CH505M5 N197D mRNA-gp160, CH505 TF mRNA-gp160	LNPs	I.M.	25 µg, 50 µg, 100 µg , 150 µg	0, 8, 16, 24
23	Phase I	NCT03934541	Peptide liposome	MPER-656 peptide liposomes	Alum	I.M.	500 µg, 2,000 µg	0, 2, 6, 12

^*a*^ The data are from ClinicalTrials.gov. R.O.A., route of administration; I.M., intramuscular; S.C., subcutaneous; Env, envelope; MPLA, monophosphoryl lipid A; GLA, glucopyranosyl lipid A; SE, stable emulsion; VPs, virus particles; N/A, not available.

## CONSENSUS-BASED VACCINE DESIGN

An alternative strategy to tackle the extensive diversity of HIV-1 is the use of consensus envelope sequences designed to minimize the genetic distance between circulating strains (Fig. 2B). Consensus viral envelope sequences may be particularly effective at eliciting cross-neutralizing antibody responses, as they tend to be closer to circulating isolates than individual strains are to each other (101). Early work started with CON-S, a consensus Env with shortened variable loops based on group M sequences, which produced sporadic heterologous nAb responses in guinea pigs and rhesus macaques, laying the groundwork for consensus-based immunogens (102, 103). A group M consensus immunogen ConM was shown to induce strong autologous nAb responses and occasional Tier 1 heterologous responses in rabbits and macaques (104). The soluble ConM SOSIP trimer exhibits high structural similarity across multiple clades and can engage early precursors of V2 apex bnAbs. Rutten et al. constructed ConC SOSIP, a clade C-specific consensus trimer, by substituting rare strain-specific residues with most conserved ones across clades, to favor cross-reactive over strain-specific antibody responses (71). In another study, Shattock and colleagues reported both soluble ConSOSL UFO.664 and membrane-bound ConSOSL UFO.750 Env trimers based on a clade C consensus design. Immunogenicity studies revealed that the soluble trimers (ConSOSL UFO.664) elicited strong autologous Tier 2 and heterologous Tier 1 neutralizing responses compared to their membrane-bound counterparts (ConSOSL UFO.750) (105). Despite these advances, further optimization of consensus-based immunogens is still needed to enhance cross-neutralization, a key goal in HIV-1 vaccine development.

## EPITOPE-FOCUSED VACCINE DESIGN

It has been observed that HIV-1 Env immunogens, at times, elicit antibody responses against non-neutralizing epitopes, which can divert the immune response away from protective neutralizing targets (106, 107). To overcome this, researchers have explored epitope-focused vaccination strategies that aim to direct the immune response toward the most relevant neutralizing regions while avoiding off-target responses. Following this approach, Wang et al. developed a three-component immunogen that includes a 33-amino-acid V3 glycopeptide epitope, a P30 T-helper sequence to enhance T cell-assisted antibody production, and the lipopeptide Pam3CSK4, for effective presentation to antigen-presenting cells (108). Immunization experiments showed that this V3-glycopeptide immunogen elicited antibodies able to recognize a variety of HIV-1 gp120 proteins; however, these antibodies were unable to neutralize the virus, likely because they had not undergone sufficient somatic hypermutation (SHM). Building on chimpanzee SIV Env studies, Andrabi et al. designed the MT145K Env trimer to specifically engage V2-apex bnAbs and their precursors that was found to be effective in priming the corresponding B-cell lineages in rhesus macaques. This trimer serves as a potential vaccine candidate to be included in sequential immunizations to elicit V2-apex bnAbs (109).

In another approach, VRC34.01 bNAb-targeted scaffolds were used, which incorporate the eight N-terminal amino acids of the gp41 fusion peptide (FP8) covalently linked to keyhole limpet hemocyanin (KLH) to enhance immunogenicity. Immunization of mice with the FP8-KLH scaffold elicited antibodies capable of cross-neutralizing approximately 20% of the global HIV-1 virus panel. Subsequent boosting of the mice with DS-SOSIP trimers carrying the relevant glycans further expanded the neutralization breadth (110). The same immunization strategy in non-human primates (NHPs) produced similar fusion peptide-directed bNAb responses, demonstrating the potential translatability of this approach. A limitation, however, is that epitope-focused vaccines are generally restricted to simple linear sequences or contiguous neutralizing determinants, which may limit their ability to induce broader antibody responses.

## MULTIVALENT VACCINE DESIGN

HBV and HPV multivalent vaccines demonstrated that presenting multiple copies of an antigen can significantly strengthen the immune response (111, 112). Nanoparticles (NPs), which are non-replicating structures ranging from about 1 to 1,000 nm, can be generated by chemical synthesis or recombinant techniques (113). Presenting antigens on the NPs allows them to interact more effectively with antigen-presenting cells (APCs), supporting B cell activation and maturation (Fig. 2D) (114, 115). Studies have shown that displaying Env in a dense, repetitive pattern on these platforms tends to produce stronger and longer-lasting antibody responses (116–118). In the following sections, we discuss how diverse forms of HIV-1 Env NPs can be leveraged to improve the quality and durability of the immune response.

Liposome-based NP platforms have been shown to enhance both the magnitude and quality of antibody responses. Stabilized synthetic liposomes displaying BG505 MD39 gp140 have been shown to augment germinal center and antigen-specific T follicular helper cell responses, eliciting higher-avidity serum IgG than soluble MD39 trimers in mice (116). Lipid-based peptide vaccines incorporating fusion peptide–MPER hybrid immunogens have the potential to elicit immune responses against membrane-active domains (119). In a recent study, Wyatt et al. demonstrated that immunization of NHPs with heterologous HIV-1 Env trimers covalently coupled to liposomes elicited cross-neutralizing serum antibody responses, highlighting the potential of liposome-based multivalent Env display in broadening neutralization breadth (120). However, liposomes relying on non-covalent His-tag/metal chelation may be less stable in serum, resulting in partial dissociation of Env trimers. More recent studies have mitigated this limitation by employing covalent attachment strategies, such as maleimide–thiol coupling, as well as optimized chelation chemistries, that markedly improve trimer retention on liposomes and preserve particle integrity *in vivo* (121).

Self-assembling protein NPs offer a robust platform to study multivalent vaccines, overcoming the need for complex purification steps and can be classified further into *in vivo* and *in vitro* assembling NPs (122). Self-assembling lumazine synthase NPs have been designed to present 60 copies of the engineered gp120 outer domain (eOD-GT6), enabling the activation of both germline and mature VRC01-class B cells (83). Ferritin-based *in vivo* NPs displaying eight copies of BG505 SOSIP.664 trimers have been shown to elicit significantly stronger antibody responses in mice and rabbits compared to soluble trimers, while maintaining proper bNAb binding; although on antigenic profiling, steric hindrance in recognition of the interface bnAbs was observed (118). The V1V2, gp120, and diverse forms of stabilized gp140 trimer based on ferritin and a 60-meric E2p that displays 20 spikes have shown to be presented in native-like trimeric conformations on NPs and are potential multivalent immunogens for HIV-1 vaccine development (123). While ferritin-based NPs offer a well-defined multivalent scaffold, they have certain limitations. These include a restricted number of displayed HIV-1 Env trimers (up to eight per particle), plausible incorporation of misfolded or non-native like trimers during *in vivo* assembly, and inefficient furin-mediated Env cleavage, which may skew the resulting nAb responses toward Tier-1 viruses (104, 118, 124). The two-component *in vitro* assembling NPs, such as I53-50 and dn2 & dn5, permit independent expression and purification of subunits that self-assemble into ordered particles in a dense array displaying 20 SOSIP trimers (117, 125). Two-component NP-based trimers showed 40-fold higher nAb responses compared to soluble trimers, are efficiently cleaved by cellular proteases, and enabled immunoaffinity selection of native-like trimers prior to assembly (126). Despite this improvement, one issue with such NPs is that the scaffold itself can trigger an immune response, which may compete with or distract the immune system from generating antibodies targeting the displayed Env protein (127). Inefficient furin-mediated Env cleavage, presenting Env trimers to a rigid NP scaffold, has been observed to sterically occlude key epitopes or impose conformational constraints that reduce the accessibility of neutralizing epitopes (104, 128). While self-assembling NPs clearly hold the potential to enhance vaccine effectiveness, further studies are warranted to refine their design and application to ensure both safety and strong immunogenicity.

## LINEAGE-BASED VACCINE DESIGN

Most current HIV-1 vaccine approaches induce only strain-specific nAbs with limited cross-reactivity. However, about 20% of infected individuals eventually develop bnAbs. Mapping the co-evolution of the bnAbs with the viruses provides a blueprint for vaccine design (9, 129). The bnAbs generally arise after prolonged virus–host antibody coevolution, where early autologous neutralizing responses gradually mature into broader activity (130). Lineage-based vaccine strategies aim to replicate this process by identifying the transmitted founder (T/F) virus and the precursor B cells that give rise to bnAb lineages. Lineage-based vaccine strategies employ gradual introduction of the immune system to a series of evolving Env variants, to mimic natural co-evolution and direct antibody responses toward defined bnAb epitopes. The first such co-evolutionary model came from donor CH505, where CH505 T/F gp120 immunogens were designed to prime precursor B cells and guide them toward bnAb maturation (Fig. 2C). This approach led to the development of two CD4 binding site-directed bnAb lineages, CH103 and CH235 (130, 131). These antibodies achieved breadth with relatively modest SHM compared to other bnAbs, relying, in part, on long CDRH3 loops (132). When tested in macaques, most CH505 Env regimens failed to elicit consistent tier-2 autologous or heterologous neutralization (133, 134). However, an MVA-vectored, CH505 transmitted founder CH505 Env prime-boost approach showed modest ability to induce autologous neutralization (135). Vaccine-induced bnAb responses hence require lineage-based immunogens, designed to select for improbable mutations, as shown by Saunders et al. A modified Env immunogen, CH848 10.17DT was designed and engineered with V1 glycan deletions that showed high binding affinity to bnAb precursors of either a CD4 binding site (CH235) or V3-glycan (DH270) bnAb lineage with acquired improbable mutations. These immunogens were found to elicit serum neutralizing antibodies in human bNAb precursor knock-in mice and NHPs (136). In a recent study, immunization of macaques with the engineered Env CH505 M5.G458Y carrying N279K and G458Y substitutions demonstrated induction of CD4-mimicking, CD4bs antibodies that resembled human VH1-46-like bnAb precursors (137). In a recent study, mutation-guided clonal lineage design was explored based on MD simulations and structural modeling to map how bnAbs initially engage the HIV-1 Env through transient encounter states before forming stable complexes. A modified Env immunogen CH848.d949.10.17 was designed that conferred affinity gains against V3 and CD4bs bnAb lineages and successfully steered antibody maturation *in vivo* (138).

In a study carried out by Doria-Rose et al. to delineate the development of the V1V2-directed VRC26 lineage in the CAP256 donor, characterization of the antibody evolution and co-evolving viruses revealed that early development of V1V2-directed nAbs can be achieved by initial activation of B cells with a long CDRH3 and limited SHM, to evolve into bnAbs (Fig. 2C) (139). Although the highly variable V1V2 region usually drives strain-specific responses, the CAP256-VRC26 antibodies stand out because they recognize semi-conserved elements within the V2 region and offer critical insights for vaccine design (140). Several V1V2-directed bnAbs have been isolated from other donors, such as PG9/PG16, PGT145/PGDM1400, and CH01–04, but CAP256 remains the sole donor studied longitudinally from early infection, defining a developmental roadmap for this lineage of bnAbs (89, 90, 141, 142). Viral sequencing revealed that recombinants of primary and superinfecting viruses could engage the unmutated common ancestor (UCA) of the lineage and initiate B cell activation (143). Phylogenetic analyses showed two evolutionary outcomes: one branch that failed to overcome viral escape (‘dead-end’ antibodies) and another that produced either highly mutated but narrow ‘off-track’ antibodies or bnAbs capable of recognizing diverse variants (140, 143). These findings highlight the value of preserving native Env trimer structures in immunogen design to guide similar bNAb lineages and underscore the need to define the specific viral triggers that launch such exceptional immune responses.

## VECTOR-BASED VACCINE DESIGN

Vector-based HIV-1 vaccine design is based on engineered viral vectors to deliver antigens, with the aim of inducing strong cellular and humoral immune responses. In this approach, the vectors are genetically modified to safely express HIV-1 proteins, to prime the immune system to generate a robust immune response. Over several years, vectors have been explored for safe human use, including adenoviruses (AdV), modified vaccinia Ankara (MVA), chimpanzee adenovirus Oxford 1 (ChAdOx1), and canarypox virus which has been extensively studied in both preclinical models and clinical trials. For instance, the RV144 trial employed a canarypox-based vector (ALVAC) expressing HIV-1 genes combined with a gp120 protein boost (AIDSVAX), resulting in modest efficacy and providing important insights into immune correlates of protection (144, 145).

A significant limitation of these platforms stems from pre-existing immunity, as humans frequently harbor adenoviruses and mount immune responses against specific adenovirus serotypes (146). Notably, pre-existing immunity to AdV5 necessitated the premature termination of the STEP clinical trial, after it was observed that AdV5-seropositive participants exhibited an increased susceptibility to HIV-1 infection (147). Recently, in the HIV-CORE 006 trial, a mosaic T-cell vaccine combining ChAdOx1 and MVA was found to be safe and triggered strong immune responses in healthy adults (148). In the DNA-based vaccine approach, HIV-vectors encoding HIV-1 antigens delivered plasmid DNA directly into host cells, where their expression triggered immune responses against the viral antigens. However, in the HVTN 505 trial, the DNA/rAd5 vaccine did not reduce HIV-1 acquisition or affect viral-load set points (149). These findings warrant the need for alternative vaccine strategies to elicit more effective protective immune responses. Despite promising preclinical data, clinical trials have produced mixed outcomes, highlighting the need to optimize vaccine vectors and the immunization schedules to achieve effective and durable protection against HIV-1.

## STRUCTURE-GUIDED VACCINE DESIGN

Structure-guided vaccine design has transformed the field of viral immunogen design by enabling rational stabilization of class I fusion proteins in their native prefusion conformations, thereby eliciting protective nAb responses (Fig. 2G). This approach addresses the intrinsic metastability of these viral proteins and has been successfully applied to several major pathogens. In the case of SARS-CoV-2, cryo-EM studies of the spike glycoprotein enabled the introduction of two proline substitutions S “2P” (K986P, V987P) that stabilized the prefusion spike and formed the basis of all first-generation COVID-19 vaccines (150, 151). Similarly for RSV, McLellan and colleagues solved the high-resolution structure of the prefusion RSV F glycoprotein, revealing a potent neutralizing epitope at the trimer apex and enabling the design of RSVPreF immunogens that dramatically enhanced immunogenicity evaluated in animal models and clinical trials (152).

In HIV-1, the first near-native, cleaved, soluble Env trimer structure (BG505 SOSIP.664) by Julien et al. and Lyumkis et al. provided a structural template for Env stabilization and vaccine design (16, 18). Structure-guided mutational strategies subsequently yielded numerous stabilizing modifications. Guenaga et al. described the TD8 mutations, which increased thermostability and improved trimerization across diverse clades while incorporating further additional TD12, TD14, TD15 mutations (68). Env stabilization strategies include the introduction of additional disulfide bonds I201C–A433C in BG505 DS-SOSIP to prevent CD4-triggered opening (153), to stabilize the trimer in prefusion conformations. The Trimer 4571 (DS-SOSIP) was tested in a Phase I clinical trial and was found to be safe and immunogenic in the participants (72, 154). Structure-guided point mutations such as E64K, A316W, S306L, and R308L, aimed to reduce exposure of V3/CD4i-directed non-neutralizing epitopes and glycan engineering to mask immunodominant sites, and played a major role in the Env stabilization (66). Germline-focused immunogens, such as MD39-10MUT/11MUTB, mark an important step forward, using structure-guided design to better engage the precursors of inferred bNAb precursors (69). In another study, structure-guided redesign of the metastable HR1 region in the UFO (Uncleaved Prefusion-Optimized) addressed trimer folding and achieved improved yields of homogeneous prefusion trimers (155). Andrabi and colleagues recently reported a structure-guided stabilized Env construct, CAP256.SU.SCT, which employs a combinatorial mutational strategy incorporating elements from DS-SOSIP, NFL-TD8, and RnS designs to stabilize the V2 apex of Env. Detailed structural analysis and antigenicity profiling of the construct suggest the suitability of the SCT platform for vaccine design (156). Together, these findings highlight how insights from structural biology, paired with protein engineering, can accelerate the development of HIV-1 vaccines.

## RECENT DEVELOPMENTS AND CLINICAL TRIALS

Over the past decade, the viral vaccine field has witnessed significant technological advancements. With the success of SARS-CoV-2 mRNA vaccines, researchers are now adapting this platform for HIV-1 immunization (Fig. 2F). The design of mRNA-based HIV-1 vaccines has emerged as a promising strategy to address issues such as rapid viral mutation and immune evasion mechanisms (157). Recent efforts have focused on optimizing mRNA constructs to encode stabilized forms of the HIV-1 envelope glycoprotein (Fig. 2F) (158, 159). Computational studies have developed algorithms to increase stability and half-life of mRNA vaccines so as to generate robust immune responses *in vivo* (160).

In a recent study, Schief and colleagues demonstrated that immunization with mRNA-encoded membrane-bound version of the stabilized Env trimer (BG505 MD39.3) elicited stronger neutralizing antibody responses, as compared to its mRNA-encoded soluble env (159). In a recently conducted HVTN 302 Phase I, open-label, randomized, multicenter trial, three mRNA-encoded Env trimer vaccines MD39.3 gp151, MD39.3 gp151 CD4KO (membrane bound), and MD39.3 gp140 (soluble trimer) were evaluated. Three immunizations with these membrane-anchored mRNA-encoded trimers elicited autologous tier 2 serum nAbs in 80% of vaccinees in contrast to only 4% of the vaccinees receiving the soluble trimer (158). The immune system is trained in phases by sequential mRNA vaccines, which progressively direct B cells to mature into bnAbs. Almost all subjects (97%) in the Phase I studies displayed the anticipated initial activation of naive B cells, paving the way for booster shots to further mature these cells into plasma cells that can produce bnAbs (161). Despite these developments, there are still difficulties in refining mRNA vaccine formulations to reduce side effects and guarantee long-term effectiveness (162). Nevertheless, the development of mRNA-based vaccines is a major step in the direction of a successful HIV-1 vaccine.

The TTT-platform and env gp151 design are recent developments in HIV-1 vaccine research. By genetically joining three Env protomers into a single construct, the Triple Tandem Trimer (TTT) platform is a neo HIV-1 vaccine design. This design offers incorporation of protomers from diverse HIV-1 strains (163). By preserving the gp41 HR2 and exposing additional MPER-specific epitopes, the gp151 Env design offers better design over the gp140 to improve trimer stability and immunogenicity (164). A recent study showed that the germline-targeting 3nv.2 trimer, designed to engage multiple bnAb precursors, elicited potent neutralizing responses in mice and rhesus macaques, combining the strengths of its parent immunogens and guiding future germline-based immunogen design (165). Preclinical research revealed that when administered in heterologous prime-boost regimens, gp151-based vaccines induced autologous tier 2 nAbs and lowered the viremia during SHIV challenge (166). A recent clinical trial using a DNA and replicating vaccinia virus vector expressing CN54 gp151 Env showed the formation of anti-gp120 IgG and polyfunctional CD4^+^ T cell responses, thus supporting the development of gp151-based platforms (167).

Recent clinical trials highlight significant advances in vaccine design and delivery strategies, with the goal of eliciting protective neutralizing response (168). Most trials are based on gp140-based Env trimers (Table 1) that maintain native-like conformation critical for effective neutralization. In the HVTN 133 Phase I trial, an MPER peptide-liposome immunogen targeting the unmutated ancestor (UCA) of an MPER bnAb elicited polyclonal MPER-directed antibodies in humans, with the most potent lineages neutralizing diverse HIV-1 strains and acquiring essential functional improbable mutations (169). In the IAVI G001 Phase I clinical trial, the eOD-GT8 NP was immunized and showed polyfunctional CD4 T-cells, specific for eOD-GT8 in 84% of the vaccinees (87). In a recent Phase I clinical trial, the Env trimer BG505 SOSIP.v4.1-GT1.1 elicited VRC01-class bnAb precursor responses at high frequency in most vaccine recipients; the isolated monoclonal antibodies from these individuals neutralized wild-type pseudoviruses and exhibited structural features similar to the bnAb VRC01 (170). Next-generation designs, including mRNAs encoding germline immunogens eOD-GT8 60mer, Core-g28v2 60mer, and gp150 based envelope N332-GT5 gp151, are being explored to improve immunogenicity and achieve neutralization breadth (NCT06694753) (171). Combinatorial prime-boost regimens are being explored by integrating DNA, viral vectors, and protein immunogens, to enhance antibody breadth and durability (NCT04844775) (172, 173). These studies underscore the field’s evolution toward new vaccine design and delivery platforms, along with structure-guided stabilization of envelopes, bringing the goal of an effective HIV-1 prophylactic vaccine closer to reality and to eliminate HIV-1 infection.

## CONCLUDING REMARKS

HIV-1 remains a formidable challenge in vaccinology due to its extraordinary ability to evade the immune system. These challenges have driven the researchers to develop new strategies, combining existing approaches or adopting the concepts from other fields of vaccinology. Pre-exposure prophylactic drugs (PrEP) are effective in preventing HIV-1 infection in high risk, exposed seronegative individuals. In resource-limited settings, inadequate adherence to the treatment, insufficient awareness, and suboptimal clinical management of adverse effects significantly hinder efforts to combat HIV-1 infection (174). In recent years, with the availability of high-resolution structures of native-like trimers and recently identified bnAbs, along with detailed information on their developmental pathways, enhanced our understanding of the host immune response to HIV-1 and has propelled the field of HIV-1 vaccinology forward. This review provides insights into the diverse strategies that could be employed to recapitulate natural infection, through a coordinated vaccination regime, to efficiently induce heterologous nAbs. The development of stabilized env trimers capable of inducing autologous Tier-2 nAb responses consistently in different animal models reflects their potential as promising vaccine candidates. Encouragingly, some of these candidates are being evaluated for immunogenicity in ongoing Phase I/II clinical trials (referring to Table 1). However, in a few of the studies, these immunogens either failed to induce a cross- neutralizing response or induced sporadic heterologous Tier-2 responses; therefore, an alternative strategy or integration of distinctive strategies such as multivalent and lineage-based platforms is desired to efficiently induce heterologous nAbs. One strategy involves priming with germline-targeting immunogens strategically designed to expand rare bnAb precursors, followed by boosting with lineage-based immunogens derived from patient or consensus sequences to guide the antibody response toward a specific bNAb epitope. An avenue toward eliciting robust and sustained bNAb protective responses has yet to be achieved and important knowledge gaps remain to be yet addressed.

## BEST PATH FORWARD FOR HIV-1 VACCINES

In the coming years, HIV-1 vaccine research is expected to focus on multi-component strategies that combine different platforms to stimulate bnAbs while also supporting long-lasting T-cell immunity. The strategy usually starts with germline-targeting immunogens designed to activate the rare precursor B cells that can evolve to give rise to bnAbs. This is followed by a series of carefully guided boosts that gradually reintroduce glycans and structural features, steering B cells toward the right maturation pathway. In the later stages, stabilized, native-like trimers displayed on NPs help reinforce glycan tolerance and expand the breadth of neutralization. Most of the vaccines currently under evaluation target HIV-1 Clade A and show limited effectiveness against Clade C viruses, which make up more than half of the global infections. To better tackle the global diversity of HIV-1, future vaccines are likely to combine consensus or mosaic sequences from diverse Clades and CRFs into multicomponent Env-based formulations. Integration of T-cell epitopes with the currently available vaccines will provide cellular immunity and enhance bnAb responses. Artificial intelligence (AI) and computational biology can aid in accelerating HIV-1 vaccine design by predicting optimal Env immunogens, guiding germline-targeting strategies, and simulating immune protective responses. Integrating AI with structural and immunological data offers a powerful approach to develop next-generation vaccines that can elicit broad and potent neutralizing antibody responses that confer protection (175). Ongoing clinical trials, paired with information gained from B-cell repertoire analysis and structural studies, will guide the selection of boosters and optimize vaccine regimens. Population level B-cell genetics can further inform prime-boost strategies. Ultimately, optimal integration of diverse vaccine strategies offers the most promising way to generate strong and durable anti-HIV-1 immune responses and move closer to the long awaited goal of an effective HIV-1 vaccine.
